# Ti_2_N nitride MXene evokes the Mars-van Krevelen mechanism to achieve high selectivity for nitrogen reduction reaction

**DOI:** 10.1038/s41598-021-04640-7

**Published:** 2022-01-13

**Authors:** Denis Johnson, Brock Hunter, Jevaun Christie, Cullan King, Eric Kelley, Abdoulaye Djire

**Affiliations:** 1 Artie McFerrin Department of Chemical Engineering, Texas A&M University, College Station, TX 77843 USA; 2 Department of Chemical Engineering, Auburn University, Auburn, AL 36849 USA; 3 Department of Chemical Engineering, Prairie View A&M University, Prairie View, TX 77446 USA; 4 Department of Mechanical Engineering, Prairie View A&M University, Prairie View, TX 77446 USA; 5 Department of Materials Science and Engineering, Texas A&M University, College Station, TX 77843 USA

**Keywords:** Materials for energy and catalysis, Electrocatalysis, Electrochemistry, Nanoscale materials, Two-dimensional materials, Hydrogen fuel, Catalysis, Electrochemistry, Energy, Green chemistry

## Abstract

We address the low selectivity problem faced by the electrochemical nitrogen (N_2_) reduction reaction (NRR) to ammonia (NH_3_) by exploiting the Mars-van Krevelen (MvK) mechanism on two-dimensional (2D) Ti_2_N nitride MXene. NRR technology is a viable alternative to reducing the energy and greenhouse gas emission footprint from NH_3_ production. Most NRR catalysts operate by using an associative or dissociative mechanism, during which the NRR competes with the hydrogen evolution reaction (HER), resulting in low selectivity. The MvK mechanism reduces this competition by eliminating the adsorption and dissociation processes at the sites for NH_3_ synthesis. We show that the new class of 2D materials, nitride MXenes, evoke the MvK mechanism to achieve the highest Faradaic efficiency (FE) towards NH_3_ reported for any pristine transition metal-based catalyst—19.85% with a yield of 11.33 μg/cm^2^/hr at an applied potential of − 250 mV versus RHE. These results can be expanded to a broad class of systems evoking the MvK mechanism and constitute the foundation of NRR technology based on MXenes.

## Introduction

The large-scale production of ammonia (NH_3_) is considered to be one of the most crucial achievements in recent history, and is responsible for more than doubling the carrying capacity of our society. NH_3_ is a crucial chemical and feedstock for our current society, and is used in the production of fertilizers, explosives, fibers, plastics, and many more^1^. This use as a fertilizer accounts for more than 80% of current NH_3_ consumption worldwide, and is the reason that we, as a race, are able to achieve the population levels that we currently maintain^2^. To achieve the level of NH_3_ production necessary for this, the Haber Bosch (HB) process was patented in the early 1900’s, and has remained relatively unmodified to this day. The HB process works by reacting atmospheric nitrogen (N_2_) with hydrogen gas (H_2_) at high temperatures and pressures (600 °C and 200 atm) to produce NH_3_^3^. The two main drawbacks to the HB process are (1) the high temperature and pressure necessary for the reaction to take place, which causes a large energy footprint for the process; (2) the majority of the hydrogen feedstock comes from non-renewable sources such as steam reforming of natural gas^4^. This results in an environmentally damaging process where approximately 2 equivalents of carbon dioxide (CO_2_) are released into the atmosphere for every 1 equivalent of NH_3_ that gets produced^5^. For these very reasons, it is imperative to find a replacement, or complementary, process to the traditional HB process, and one such possibility comes in the form of the electrochemical nitrogen reduction reaction (NRR).

The electrochemical NRR benefits from (1) the protons needed to form NH_3_ come from a renewable source such as water; (2) the reaction occurs at ambient temperature and pressure allowing for lower energy and smaller equipment costs^6^. In order for the electrochemical NRR technology to be economically viable, and perhaps, replace and/or complement the HB process, the process must exhibit a high selectivity, referred to as Faradaic efficiency (FE) toward NH_3_ over other reactions, notably, the hydrogen evolution reaction (HER)^7^. This requires an efficient catalyst and electrolytic system. Recently, great effort has gone into finding efficient catalysts for NRR^8^. Some of these catalysts include single atom catalysts^9^, pure metal hybrid clusters^10,11^, transition metal carbides^12^, oxides^13^, nitrides^14^, or sulfides^15^, perovskites^16^, titanium nanoparticles^17^, and polyhedral copper superstructures^18^.

For most catalysts, the typical mechanism for NRR is either an associative or dissociative mechanism, which involves the adsorption of N_2_ to the surface followed by weakening of the N≡N triple bond, which takes a large amount of energy. Once the triple bond is weakened, then proton/electron (H^+^/e^−^) transfer steps slowly form NH_3_, which then desorbs from the surface and is released into the electrolyte^19^. Compared to the conventional associative or dissociative mechanism, the MvK mechanism follows a lower energy pathway that allows for higher production rates from nitrogen-containing catalyst structures. The MvK mechanism is able to access this lower energy pathway because the produced NH_3_ originates from the nitrogen atoms within the catalyst structure. These nitrogen atoms react with protons from the electrolyte to form N–H bonds, followed by dissociation and diffusion of NH_3_ from the catalyst structure to the electrolyte. The vacancy that is thus formed from this dissociation is replenished by gaseous N_2_ allowing for the cycle to continue without the need for regeneration time or large energy input to break the N≡N bond^14^. For this reason, it is believed that nitride catalysts provide the most favorable pathway for the realization of the NRR technology^20^.

When compared to bulk nitrides, two-dimensional (2D) nitride materials, known as MXenes, are more favorable for the MvK mechanism due to their high electrical conductivity, high surface-area to volume ratio, and facile access to the N sites^21–24^. However, there exists no experimental work on nitride MXene NRR or their ability to evoke the MvK mechanism.

Herein, we characterize and use a Ti_2_N nitride MXene as an efficient electrocatalyst for the conversion of N_2_ to NH_3_ in a 0.1 M hydrochloric acid (HCl) electrolyte. Characterization data, including X-ray diffraction (XRD), Raman, UV–Vis, and scanning electron microscopy (SEM), provides powerful proof for successful etching of the parent MAX phase to form MXene. With XRD, an interlayer spacing of 0.920 nm was found, and SEM showed a lateral flake size of roughly 5 μm, indicating the 2D nature of the catalyst. With this nitride MXene catalyst, we achieved a yield of 11.33 μg/cm^2^/hr with a FE of 19.85% towards NH_3_ at an applied potential of − 250 mV versus RHE. When compared against carbide and carbonitride MXenes, and other pristine transition metal-based catalysts, this nitride MXene vastly outperformed in terms of both activity and selectivity. Through preliminary data involving Ar-saturated electrolyte, we lay the footstones to further the hypothesis of the MvK mechanism playing an important role in achieving this enhanced performance.

## Results and discussion

**Figure 1:**
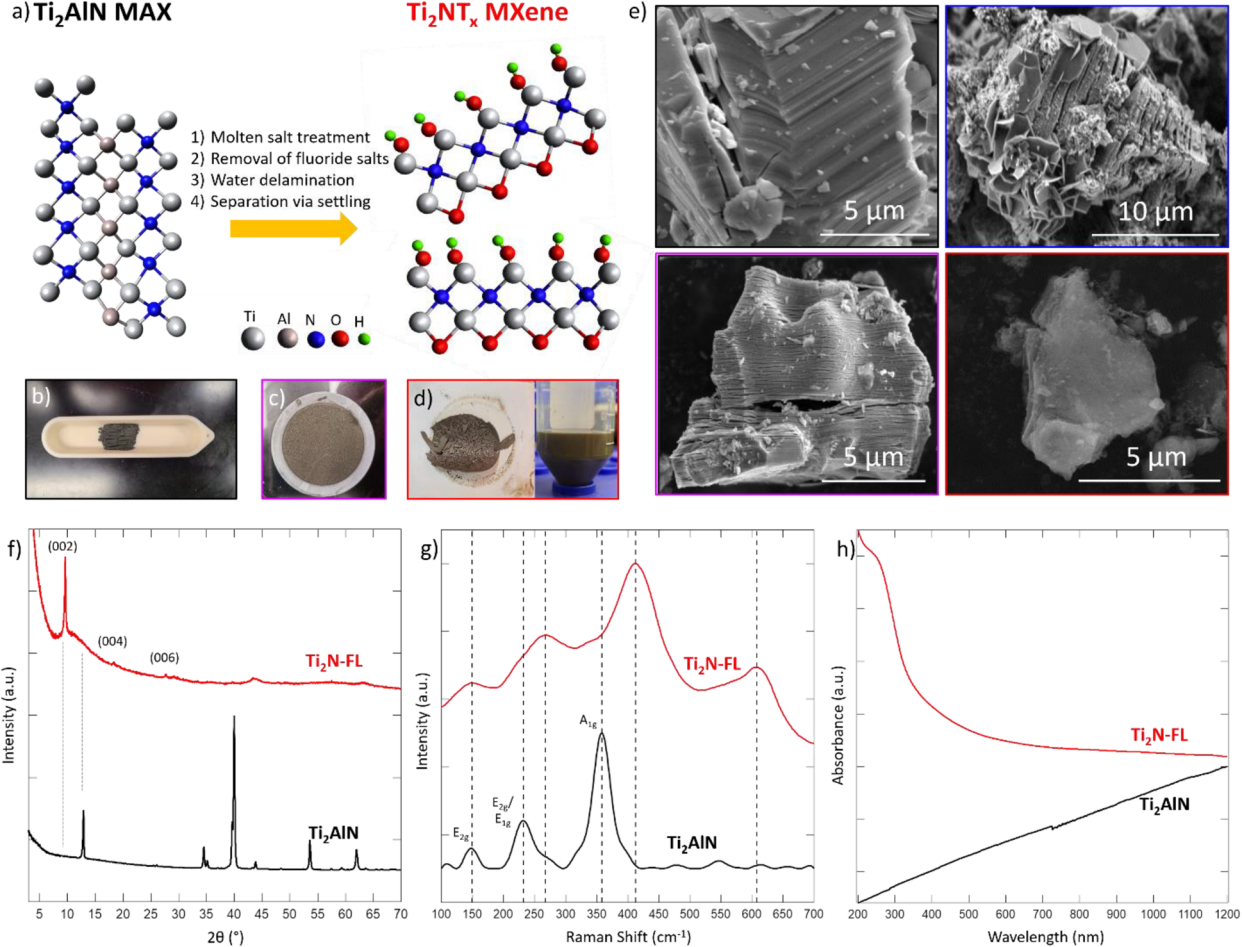
(**a**) Schematic illustration of the synthesis of Ti_2_NT_x_ MXene via oxygen-assisted molten salt fluoride treatment of the parent MAX phase Ti_2_AlN at 550 °C for 5 h under flowing argon, then exposure to air, followed by fluoride salt removal in 4 M H_2_SO_4_, finally delamination is accomplished via sonication in water for 4 h. Models are not based on gathered data, only as a general guideline. In lab photographs of (**b**) MAX phase, (**c**) Multilayer Ti_2_N MXene after acid washing, and (**d**) Single layer MXene acquired after delamination in water. (**e**) SEM imaging of Ti_2_AlN MAX phase (black outline), molten salt treated MAX phase (blue outline), multilayer Ti_2_N MXene (purple outline), and few layer Ti_2_N MXene (red outline). The lateral size of the individual MXene flakes is roughly 5 μm. (**f**) XRD, (**g**) Raman, and (**h**) UV–Vis spectra of Ti_2_AlN MAX phase (black) and single layer Ti_2_N MXene (red). XRD was gathered using a zero-diffraction silicon plate with a well. Raman spectroscopy was gathered using 532 nm laser at 5% power at a 1 s exposure time. UV–Vis spectroscopy was collected using water as the matrix.

Due to the challenges in their synthesis, a great amount of work was done to assure a successful synthesis was achieved prior to the NRR evaluation. The Ti_2_NT_*x*_ MXene was prepared from the parent MAX phase (Ti_2_AlN) via an oxygen-assisted molten salt fluoride etching technique under Ar flow, followed by exposure to air in which volume expansion occurs and color shifts from black to brown. Afterwards, the material is washed with sulfuric acid, followed by sonication in water to delaminate to single layer MXene flakes. The delamination in water is chosen to separate the exfoliated layers into a few-to-single layer morphology, which in turn will improve the catalyst’s performance in the NRR due to the increase in available active surface area. This synthesis method is summarized, and pictures of material at different steps in the synthesis are provided, in Fig. 1a–d.

### Morphological properties

It’s imperative to understand the morphological changes after the synthesis prior to the NRR characterization, as these can affect the MvK mechanism. The morphological transition from MAX to MXene due to the synthesis procedure was analyzed via SEM. Figure 1e shows the SEM images for the Ti_2_AlN parent MAX phase, molten salt treated MAX phase, multilayer Ti_2_NT_*x*_ MXene, and delaminated (or few-to-single layer) Ti_2_NT_*x*_ MXene. It is clear to see the closely-packed layer structuring of the MAX phase (Fig. 1e black outline) is opened to the expected accordion-like structure upon completion of the oxygen-assisted molten salt fluoride process (Fig. 1e blue outline). Successful removal of the fluoride salts via sulfuric acid washing is evidenced by the thinning of the individual layers (Fig. 1e purple outline), consistent with the XRD results (Fig. 1f), and the low amount of sodium and potassium present in the electron dispersive spectroscopy (EDS) analysis (Fig. S1, supplementary information). After delamination in water, single layer flakes of lateral size 5 μm can be observed (Fig. 1e red outline). This flake size is larger than other Ti_2_N MXenes synthesized before, and will be beneficial for NRR electrocatalysis^25,26^. Alongside the visible changes in morphology, EDS further corroborates the successful synthesis of Ti_2_NT_*x*_ material. The parent MAX phase has Ti:Al and Ti:N ratios of 2:1 for both, which is consistent with expected stoichiometry (Fig. S1, supplementary information). After oxygen-assisted molten salt treatment, the presence of Al is greatly reduced, while Na and K presence can be observed clearly (Fig. S1, supplementary information). The final delaminated MXene material shows a Ti:N and Ti:Al ratio of 2:1 and 18:1 or greater, respectively (Fig. S1, supplementary information). The large presence of Si in the EDS spectra is due to the substrate used for analysis. This major removal of Al from the parent structure will facilitate easier access to the N sites allowing for utilization of the MvK mechanism.

### Structural and optical properties

Prior to the NRR evaluation, extensive characterization is done on the material to ensure phase purity. Analysis of the bulk structure and composition of the material is conducted via powder XRD. Figure 1f shows the progression of the XRD patterns as the material evolved from Ti_2_AlN MAX phase (black) to delaminated single layer Ti_2_N material (red). The XRD pattern of Ti_2_AlN MAX phase matches with expectations from previous works^25,26^. After the oxygen-assisted molten salt treatment process, we observe a clear shift to lower angles and a slight broadening of the (002) peak, as well as a drastic decrease in the (104) peak centered at ~ 40°, which is indicative of successful etching of the Al atoms from the 3D MAX structure to form sheets of Ti_2_N (Fig. S2, supplementary information). Washing with sulfuric acid (Fig. S2, supplementary information) successfully removed the remaining fluoride salts on the structure as can be seen by the elimination of the peaks assigned to KF, LiF, and NaF (Fig. S3, supplementary information). Upon sonication, phase pure Ti_2_N with a clear layered structure as evidenced by the existence of the (002) peak at ~ 9.6°, followed by (004) and (006) peaks at 19° and 28.5°, respectively. This location of the (002) peak corresponds to an interlayer spacing (d-parameter) and c-parameter of 0.920 nm and 1.84 nm, respectively. In comparison, the MAX phase exhibits an interlayer spacing and c-parameter of 0.342 nm and 0.684 nm, respectively. This tripling in the interlayer spacing indicates that clear separation of the layers from the MAX phase took place, meaning that the available surface area increased, exposing more N sites, leading to enhanced NRR selectivity and activity through the MvK mechanism.

Due to the inactivity of the parent MAX phase for NRR, it is crucial to ensure that proper etching took place. To further ensure this etching took place, Raman spectroscopy was used to investigate the vibrational modes along each step of the synthesis procedure and is shown in Fig. 1g. The MAX precursor displayed three main active Raman modes (three in-plane modes and one out-of-plane mode), which is line with reported expectations of a 211-type MAX phase. The molten salt treated MAX phase (Fig. S4, supplementary information) shows a blue shift in its active modes, which can be attributed to the reduction in the crystallinity of the material as the interlayer spacing increases due to exfoliation and intercalation of salt ions. Upon acid washing, the material transitions to having four clear features at 150, 265, 400, and 610 cm^−1^ (Fig. S4, supplementary information) which are broader than the modes observed in the precursor MAX phase. This peak broadening stems from the material distortion of the P6_3_/mmc structure due to the electron density of the surface termination groups^27^. This peak broadening also supports the XRD and EDS results that more N sites will be available for the MvK mechanism. As the material exfoliation leads to separation of the layers, more H^+^ in solution will bond to the lattice N atoms more often than the termination groups, allowing for the MvK mechanism to be evoked, which will allow for higher selectivity of NRR over HER. After delamination, the four main modes are still present, with the main mode being the 400 cm^−1^ peak, corresponding to the vibration of the Ti atomic planes within the structure now that the Al layers have been removed^26^.

The acquired XRD and Raman spectra were compared against anatase, rutile, and alumina (Fig. S5, supplementary information) to confirm the stability of the newly synthesized material as no oxidation occurred during the synthesis procedure. To further confirm that no oxidation took place during synthesis, Fourier transform Infrared (FTIR) spectroscopy was taken between the MAX and MXene samples. As can be seen in Fig. S6 (supplementary information), the presence of oxygen species in the structure can be primarily attributed to surface Ti–OH groups, due to the feature at ~ 3300 cm^−1^. The Ti_2_N nitride MXene synthesized here has larger lateral size and less defects compared to the previously reported Ti_2_N nitride MXene^25,26^. This is because the new and improved synthesis approach employed here yields consistent, reproducible, and phase pure nitride MXenes as corroborated by the XRD, SEM, EDS, and Raman results. This method can be applied to more nitride MAX phases to make new nitride MXenes, that expands the application of MXene.

### Stability of the Ti_2_N nitride MXene

The Ti_2_N material shows good stability in air and in aqueous media. As stability in aqueous media is one of the requirements for a good NRR catalyst, the properties and stability of the Ti_2_N MXene was tested using UV–Vis spectroscopy (Figs. 1h, S7, supplementary information). Both the MAX precursor (Fig. 1h black trace) and molten salt treated MAX phase (Fig. S7, supplementary information) displayed purely metallic absorption properties, which is expected based on the atomic formula and reported properties of the materials^28^. Upon acid washing, the multilayer material displays absorption at 250, 500, 925, and 1175 nm (Fig. S7, supplementary information), the last three of which are attributed to multilayer absorption due to their disappearance upon delamination to few layer Ti_2_N material, which only displays an absorption peak at 250 nm (Fig. 1h red trace).

In practical applications, catalysts are expected to stay in solution for multiple days or weeks. Thus, to test whether or not the Ti_2_N catalyst will meet this expectation, a colloidal solution with water was created, and placed into a vial for long-term colloidal stability analysis. After 28 days, no precipitation of material was observed, and no significant change was observed in the UV–Vis spectrum (Fig. S8, supplementary information), vastly outperforming their carbide MXene counterparts^29^.

### NRR performance via MvK mechanism

**Figure 2:**
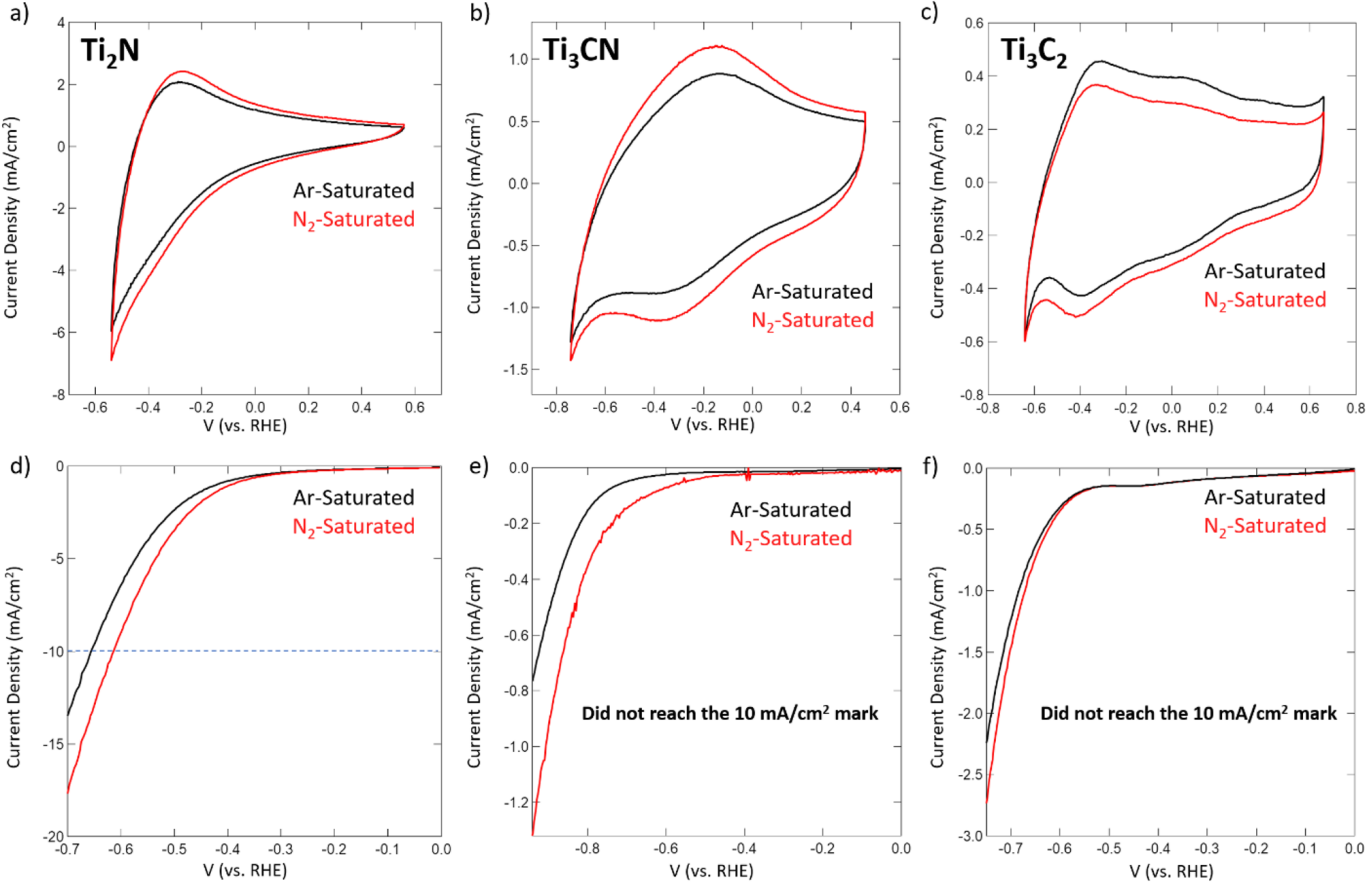
Cyclic voltammograms for (**a**) Ti_2_N, (**b**) Ti_3_CN, and (**c**) Ti_3_C_2_ MXenes conducted in Ar-saturated (black) and N_2_-saturated (red) 0.1 M HCl electrolyte. All scans conducted using a scan rate of 20 mV/s. Linear sweep voltammograms for (**d**) Ti_2_N, (**e**) Ti_3_CN, and (**f**) Ti_3_C_2_ MXenes conducted in Ar-saturated (black) and N_2_-saturated (red) 0.1 M HCl electrolyte. All scans conducted using a scan rate of 5 mV/s.

After ensuring that the synthesized Ti_2_N is phase pure and that its morphology and compositions are favorable for the MvK mechanism, we proceed to evaluate its NRR performance. We also characterize Ti_3_CN and Ti_3_C_2_ carbonitride and carbide MXene, respectively, for comparison and validation of the MvK mechanism. The catalytic NRR activity for the delaminated Ti_2_N, Ti_3_C_2_, and Ti_3_CN samples was investigated within an H-cell under ambient temperature and pressure. Figure 2 shows the cyclic voltammograms (CV) and linear sweep voltammetry (LSV) curves for these catalysts recorded in Ar- and N_2_-saturated 0.1 M HCl electrolyte. We used 0.1 M HCl as the electrolyte due to its heavy use in previously reported NRR papers, and to ensure that a steady stream of H^+^ are available for the protonation of the N_2_ molecules^8,17,18^.

When comparing between the carbide MXene and the carbonitride and nitride MXene counterparts, it is clear to see that as the ratio of N atoms to Ti atoms within the lattice structure increase, the electrocatalytic performance under NRR conditions is enhanced. While the ratio of N to Ti in the structure may not be the only factor for this enhancement (termination groups and interlayer spacing could also play a role), based on the MvK mechanism, it is reasonable to assume that a large portion of the enhancement is due to this heightened ratio. Namely, the CVs for the carbonitride and nitride grow wider when switching from Ar to N_2_, indicating more charge storage capability under N_2_-saturated electrolyte. Whereas the carbide merely experiences a downward shift of its CV while still maintaining the same area. This indicates that the nitride and carbonitride MXene materials are able to take on more current as the reaction conditions switch, which will allow for enhanced NH_3_ production. The lack of changes in the CV of the carbide MXene when switching to N_2_-saturated electrolyte provides preliminary evidence of limited performance for NRR, which is in alignment with previous NRR studies of Ti_3_C_2_^23^. In terms of the LSVs, the carbonitride and nitride experience a much greater anodic shift in their onset potential when switching from Ar-saturated to N_2_-saturated, while the carbide experiences next to no shift when switching between reactive conditions. The lack of shift in the LSV for the carbide is once again indicative of a material whose selectivity and performance towards NRR is extremely limited. The anodic shift in the LSV for the nitride and carbonitride catalysts indicate that switching to N_2_-saturated electrolyte leads to a drastic improvement in catalytic activity, due to easier NH_3_ production through NRR. It is also clear to note that the Ti_2_N material is able to display an almost ten-fold greater current density in the LSV when compared to the carbonitride and carbide. This greater current density observed for the Ti_2_N can be attributed to the greater amount of N sites and improved conductivity and reactivity^30–32^. This initial evidence of the MvK mechanism in Ti_2_N is further supported by a N_2_-free experiment, which shows that in Ar-saturated electrolyte, the Ti_2_N consistently draws moderate current densities over 50 cycles implying that some reaction could be occurring within the crystal lattice structure of the material. This cycling experiment also indicate the stability of the catalyst under these environments.

**Figure 3:**
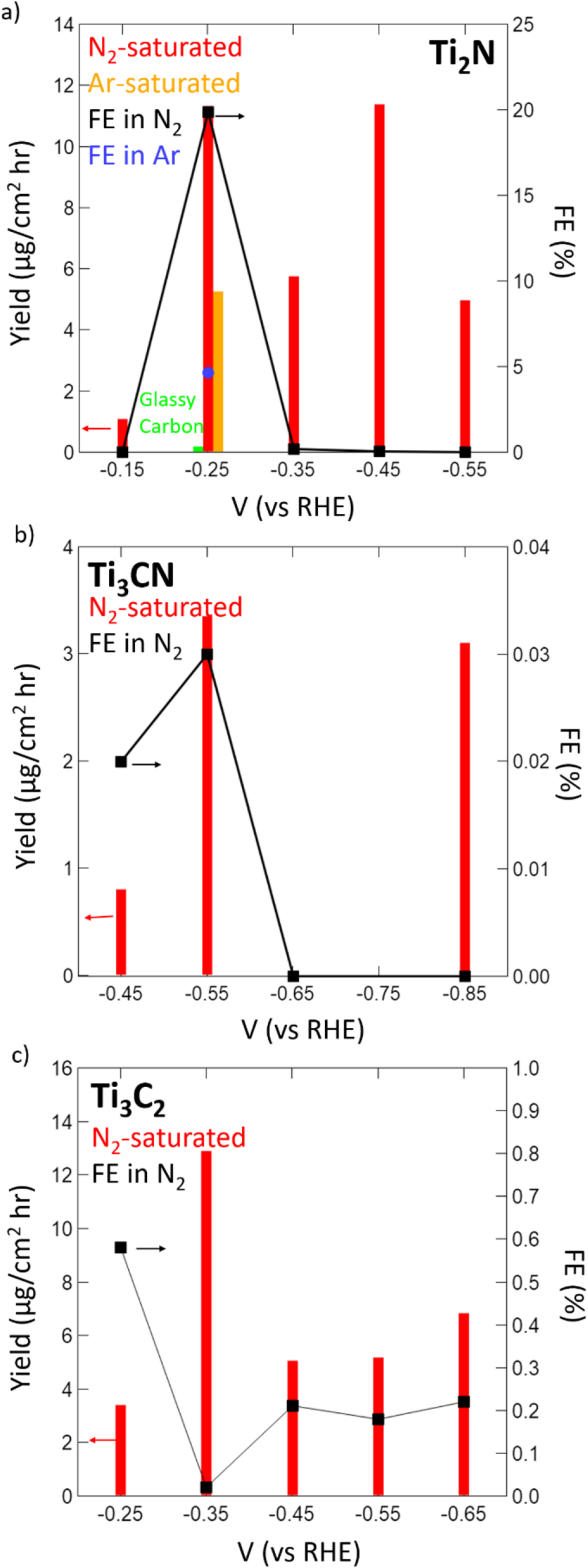
NH_3_ yield and Faradaic efficiency of (**a**) Ti_2_N, (**b**) Ti_3_CN, and (**c**) Ti_3_C_2_ MXenes at differing potentials after 4-h chronoamperometry experiments. An experiment was carried out in Ar-saturated electrolyte on Ti_2_N MXene to provide evidence of MvK mechanism. All bars correspond to yield values (read to the left) while lines with markers correspond to FE values (read to the right).

For the Ti_2_N nitride MXene, the onset potential for NRR was observed to be around − 0.25 V versus RHE, so the voltage window of catalytic performance was based around this area. Figure S9 (supplementary information) displays the time-dependent chronoamperometry (CA) data gathered during the experimental runs on Ti_2_N, Ti_3_CN, and Ti_3_C_2_, respectively. The CA curves show good stability throughout the 4-h NRR experiment. Through use of the indophenol blue method and Watt–Chrisp method, the yields of ammonia and hydrazine, respectively, were estimated. No significant yields of hydrazine were detected for any of the catalysts during any of the experimental runs (Fig. S10, supplementary information). Similar maximum absorbance values from the indophenol blue method were observed for applied potentials of − 0.25 and − 0.45 V versus RHE (Fig. 3a). Through use of the generated calibration curve (Fig. S11, supplementary information), the concentration, and therefore yield, of produced NH_3_ was estimated to be about 11.33 and 11.38 μg/cm^2^ hr, respectively. When taking into account the selectivity, or FE, of Ti_2_N towards NRR over HER, the optimal NRR experimental run was observed at − 0.25 V versus RHE with a FE of 19.85% compared to 0.03% for the − 0.45 V versus RHE run. This drastic drop off in FE occurs due to the predominance of HER at more cathodic potentials as protons are drawn more towards the termination groups that catalyze HER instead of the edge sites where the nitrogen atoms are more easily accessed^33^. This preference for HER at more negative potentials requires that these lower potentials be used for future studies. These performance metrics outperform pristine carbide MXenes^22^, ruthenium-platinum alloys^34^, and MoS_2_^35^ in both yield and selectivity; modified carbide MXenes^23^ in terms of yield; and ruthenium single atom catalysts^36^ in terms of selectivity. Extra attention should be paid to the vast improvements in FE that we observe, as this can be directly related to the MvK mechanism allowing for heightened selectivity towards NRR over the troublesome HER competition.

When comparing the catalytic capabilities of the carbide, carbonitride, and nitride MXenes, it is clear to see a large distinction between materials, especially in terms of FE. Following a similar strategy as that applied to the nitride MXene catalyst, a maximum yield and FE for the carbonitride was observed at an applied potential of − 0.55 V versus RHE, and was found to be 3.352 μg/cm^2^ hr with an efficiency of 0.03% (Fig. 3b), significantly lower than that for the Ti_2_N catalyst. The carbide MXene catalyst exhibits a maximum yield and FE of 12.90 μg/cm^2^ hr and 0.58%, respectively (Fig. 3c). Interestingly, these maximum values were observed at different potentials (− 0.35 V for yield and − 0.25 V for FE) compared to occurring at the same potential like what was observed with the nitride and carbonitride indicating that a trade-off between yield and FE could exist for the carbide due to it requiring an associative/dissociative mechanism to form NH_3_—rather than an MVK mechanism, like in the case of Ti_2_N and Ti_3_CN. While the carbide MXene maximum yield rate is slightly higher than that of the nitride, the extremely low FE towards ammonia (~ 0.02%) production significantly hinders its application in this system. Once again, this low FE occurs due to the predominance of HER at more cathodic potentials. Whereas, in the N-based catalysts, the MvK mechanism is found predominant, which suppresses the HER.

The stability of the catalyst was analyzed through long-term CA experimentation at the highest performing reaction conditions (− 250 mV), and the Ti_2_N catalyst is stable across this entire time span (Fig. S12, supplementary information). To further ensure stability of the catalyst, SEM imaging was conducted on the material before and after 4-h NRR conditions by transferring the drop-cast material to carbon tape. The layered morphology before and after are similar (Fig. S13, supplementary information), further indicating that the material is stable under the NRR conditions.

To ensure accuracy of our results and rule out the possibility of contamination in the gas inlet, several control experiments were conducted. For these experiments, blank electrodes were run through the system at experimental operating conditions, and it was found that no formation of ammonia or hydrazine took place, indicating that any product formation observed from experimental runs came solely from the catalytic MXene material present (Fig. S14, supplementary information). MXene material was then analyzed by performing 4-h CA experiments at standing potential (0.0311 V vs. RHE), and again, no formation of either product was observed, confirming that the material is not catalytically active under no applied charge. The final experiment that was performed was placing the Ti_2_N MXene material in an Ar-saturated electrolyte under an applied charge of − 0.25 V versus RHE to establish preliminary results towards the support of the MvK mechanism on this nitride catalyst. Under these experimental conditions, it was consistently observed that a noticeable yield of ammonia (5.24 μg/cm^2^ hr) was achieved. Due to the lack of nitrogen gas in the system, and confirmation that ammonia production only stems from the catalyst material, this produced ammonia could only derive from the nitrogen within the catalyst structure, which lends heavily towards the possibility of the MvK mechanism occurring. Further experimentation is currently being conducted on the MvK mechanism, including isotopic experimentation, further post-production characterization/catalyst stability, and in-situ spectroelectrochemical analyses; and will be reported at a later time. Nevertheless, the findings reported here constitute the first evidence for an MvK mechanism in Ti_2_N and Ti_3_CN catalysts; the implications of which goes beyond MXene catalysts.

## Conclusion

In this report, we synthesized Ti_2_N nitride MXene using an oxygen-assisted molten salt fluoride synthesis technique, and characterized using XRD, Raman spectroscopy, UV–Vis spectroscopy, and SEM. Using UV–Vis, we showed high stability of Ti_2_N in water-based solutions on the timescale of 28 days. Ti_2_N outperforms both Ti_3_C_2_ and Ti_3_CN for NRR, and was able to achieve a yield of 11.33 μg/cm^2^/hr with a FE of 19.85% towards NH_3_ at an applied potential as low as − 250 mV versus RHE. This is by far the highest FE reported for any pristine transition-metal catalyst. By testing the catalyst under Ar-saturated electrolyte, we were able to show preliminary results to support the possibility of the MvK mechanism as the explanation for the enhanced production rate and FE. Future work in this field will focus on performing isotope experiments to further support the MvK mechanism theory, looking at the use of other nitride MXene materials, and the use of basic and non-aqueous electrolytes to enhance performance and stability for NRR electrocatalysis.

## Experimental methods

### Synthesizing molten salt treated Ti_2_AlN MAX (Ti_2_AlN–MST)

Ti_2_AlN MAX Phase (NanoChemAZone, > 98%) was added to a 59:29:12 weight % mixture of KF (Alfa Aesar, 99%), LiF (Alfa Aesar, 325 mesh, 98.5%), and NaF (Alfa Aesar, 99%), respectively in a 1:1 weight ratio. This mixture of MAX + MSF was transferred to a mortar and pestle and ground for 5 min. The ground mixture was then transferred to a crucible boat. Tube furnace (CM Furnaces Inc. 1730-20 HT) was purged under Ar (Airgas, Ultra High Purity) for at least 10 min. The crucible boat was then transferred into the tube furnace, the furnace was sealed and Ar flow was established and checked by submerging outlet 3/16″ ID tubing in water. The furnace was heated up to 550 °C under Ar flow at a ramp rate of 10 °C/min, then held at 550 °C under Ar flow for 5 h. The Ar gas flow was then shut off, and the outlet tubing was removed from the water to expose the furnace to a gentle, natural air flow. These conditions were held for 1 h. After this time, the outlet tubing was submerged in water again, and the Ar flow re-established while the furnace quenched down to room temperature. Once the reactor reached room temperature, the reactor was opened and the material was exposed to atmospheric air for 14 h before repeating the above listed process. Once the reactor reached room temperature the second time, the Ar flow was shut off, and the crucible boat extracted from the furnace. The resulting material (Ti_2_AlN–MST) was then collected, weighed to analyze mass increase, then transferred to a vial.

### Synthesizing multilayer Ti_2_N MXene (Ti_2_N–ML)

The Ti_2_AlN–MST was acid washed in H_2_SO_4_ (Alfa Aesar, 95–98%) diluted to 4 M following the formula of 20 mL for every 1 g of Ti_2_AlN–MST according to the following. The Ti_2_AlN–MST material was massed out and ground for 5 min in a mortar and pestle. The ground material was then suspended in water and membrane filtered onto a 0.10 μm polycarbonate membrane (Whatman Nucleopore). Once the water was drained, the 4 M H_2_SO_4_ solution was poured over top of the material on the membrane and allowed to drain through. Once the H_2_SO_4_ was fully drained, then the material was rinsed with ultrapure water until pH 6 was achieved. Upon completion, the sample on membrane (Ti_2_N–ML) was collected and dried at 50 °C under vacuum overnight. The material was then collected off of the membrane and stored in a vial.

### Synthesizing few to single layer Ti_2_N MXene (Ti_2_N–FL)

The Ti_2_N-ML was mixed with enough ultra-pure H_2_O to produce a dark colored ink (typically about 15–20 mL) in a centrifuge vial, then shaken by hand for 5 min. The vial was then sonicated for 4 h, shaking by hand occasionally to keep thorough mixing. After sonication, the supernatant (concentrated MXene) was decanted into a new vial, which was left to rest for 1 h under a fume hood. This resulted in any leftover unetched MAX phase to precipitate out. The resulting supernatant, which is a brown viscous liquid, was then membrane filtered onto a Celgard 3501 membrane. The resulting film was then collected and stored in a vial for later analysis.

### Synthesizing Ti_3_C_2_ MXene

Ti_3_C_2_ MXene (NanoChemAZone 99 wt%) was intercalated with DMSO (Sigma Aldrich, 99.9%) in the ratio of 10 mL DMSO per 0.5 g of MXene by stirring covered for 18 h. After stirring, the solution was placed in a centrifuge vial, and filled with water. This mixture was then centrifuged (Thermo Scientific Sorvall ST16) at 3500 RPM (1962 RCF) for 15 min. The supernatant was then removed, the vial refilled with water, and centrifuged again at 3500 RPM for 30 min. After removing the supernatant again, the solution was again filled with water and centrifuged again at 3500 RPM for 30 more minutes. After this final removal of the supernatant, the precipitate was filtered on a Celgard 3501 membrane, and dried in a vacuum oven overnight at 50 °C.

### Synthesizing Ti_3_CN MXene

Ti_3_AlCN MAX phase (NanoChemAZone > 98 wt%) was mixed with LiF in a mass ratio of 1:2. This mixture was then immersed in 4 M H_2_SO_4_ in the ratio of 100 mL for every 1 g of MAX, and stirred for 24 h. The resulting solution was then membrane filtered and washed with ultrapure water until pH ~ 6 was achieved. The membrane with MXene on it was then dried under vacuum at 50 °C overnight. The powder was then dispersed in DMSO for delamination, which followed the same procedure as the Ti_3_C_2_ detailed above.

### Characterization

The crystalline structure of the material was analyzed using X-Ray Diffraction (XRD, Rigaku MiniFlex 6G). Surface morphology was analyzed using scanning electron microscopy (SEM, FEI Quanta 600 FE-SEM courtesy of the TAMU Microscopy and Imaging Center) equipped with an energy-dispersive X-ray analyzer (EDS), and Raman spectroscopy (Renishaw inVia Qontor) equipped with a 532 nm laser, an 1800 lines/mm grating, and a 100 × objective lens. Absorption behavior was analyzed using ultraviolet–visible light spectroscopy (UV–Vis, Shimadzu UV-3600i Plus). Reflectance measurements were conducted on a Bruker INVENIO R equipped with a diamond crystal Platinum ATR accessory.

### Electrode preparation for electrochemistry

To prepare a sample for drop casting onto 5 mm diameter glassy carbon electrode (GaossUnion Photoelectric Technology Company L-shaped GC electrode, working area of 0.196 cm^2^), 2 mg of MXene was mixed into 8 μL of 5 wt% Nafion in ethanol (Fuel Cell Store) and 200 μL of ethanol (Acros Organics, 99.5 + %), then sonicated for 30 min. 7.5 μL of this solution was then dropped onto the GC electrode and the electrode dried at 50 °C under vacuum for 30 min.

### Electrochemical measurements

All the electrochemical measurements (nitrogen reduction reaction and electrochemical characterization) were conducted in an H-cell (GaossUnion Photoelectric Technology Company). The counter electrode (graphite) was kept separated from the reference (4 M Ag/AgCl) and working electrode (GC mentioned above) via a Nafion 117 membrane (Sigma Aldrich). Before analysis, the membrane was pretreated by heating in H_2_O_2_ for 1 h, followed by in water for 1 h, both at 80 °C. All electrochemical and NRR measurements were conducted in Ar- or N_2_-saturated 0.1 M HCl aqueous solution. The potential was controlled using a Bio-Logic SP-300 potentiostat. The potentials against reference were converted to the reversible hydrogen electrode (RHE) using E (vs RHE) = E (vs Ag/AgCl) + 0.199 + 0.0591 × pH. The chronoamperometry tests were carried out at applied potentials ranging from − 0.15 V to − 0.55 V versus RHE.

### Product quantification

NH_3_ concentration was detected via UV–Vis spectrometry using the indophenol blue method. The calibration curve was generated by creating standard NH_4_Cl solutions with concentrations of 0.0, 0.05, 0.10, 0.15, 0.20, 0.30, 0.40, and 0.50 μg/mL in 0.1 M HCl. These were then mixed in the ratio of 2 mL standard solution, 1.25 mL oxidizing solution (0.625 M sodium hydroxide containing 0.36 M salicylic acid and 0.17 M sodium citrate), 0.15 mL catalyst solution (1 wt% sodium nitroferricyanide in water), and 0.075 mL oxidizing solution (4 wt% Cl of sodium hypochlorite in water), and allowed to rest for 2 h at room temperature. The solutions, which changed from yellow to green, were measured via UV–Vis spectroscopy focused at a peak centered at 664 nm. The calibration curve (Fig. S11, Supporting Information) (y = 2.5856x − 0.2191, R^2^ = 0.9982) showed good relation between absorbance and concentration through three separate calibrations. The yield rate of NH_3_ formation was calculated using the following equation

$$Yield = \frac{{C_{{{\text{NH}}_{4} {\text{Cl}}}} \times V}}{t \times A}$$

where ${\text{C}}_{{{\text{NH}}_{4} {\text{Cl}}}}$ is the measured NH_4_Cl concentration determined from the calibration curve (in μg/mL), V is the volume of electrolyte in the system (in mL), t is the chronoamperometry duration upon sample extraction (in sec), and A is the working area of the electrode (in cm^2^).

The concentration of N_2_H_4_ was estimated using the Watt–Chrisp method, and is detailed here. To create the coloring solution, 100 mL ethanol, 12 mL 34 wt% HCl, and 2 g para(dimethylamino)benzaldehyde were mixed together. For the calibration curve, 1.5 mL standard N_2_H_4_ solutions with concentrations of 0.05, 0.10, 0.20, and 0.25 μg/mL were mixed with 1.5 mL of coloring solution, and allowed to rest at room temperature for 30 min. Afterwards, the samples, which changed color from clear to yellow, were analyzed using UV–Vis spectroscopy at 456 nm. The calibration curve (Fig. S11, Supporting Information) (y = 0.6801x + 0.0044, R^2^ = 0.9996) showed good relation between absorbance and concentration through three separate calibrations.

### Determination of performance

Faradaic efficiency was calculated using the following equation

$$FE = \frac{{3 \times F \times C_{{{\text{NH}}_{4} {\text{Cl}}}} \times V}}{Q}$$

where F is the Faraday constant and Q is the total quantity of electricity applied to the system, and assuming that three electrons are needed to produce one molecule of NH_3_.

## Supplementary Information

Supplementary Information.
