# Supplementary material for: Ti2N nitride MXene evokes the Mars-van Krevelen mechanism to achieve high selectivity for nitrogen reduction reaction
Source: Sci Rep. 2022 Jan 13;12:657. doi: 10.1038/s41598-021-04640-7 (PMC8758741; doi:10.1038/s41598-021-04640-7)
Supplement: Supplementary file 1 — Supplementary Information. [file 41598_2021_4640_MOESM1_ESM.docx]

**Supporting Information**

**Ti_2_N Nitride MXene Evokes the Mars-van Krevelen Mechanism to Achieve High Selectivity for Nitrogen Reduction Reaction**

Denis Johnson^1^, Brock Hunter^2^, Jevaun Christie^3^, Cullan King^4^, Eric Kelley^1^, Abdoulaye Djire^1,5*^

^1^Artie McFerrin Department of Chemical Engineering, Texas A&M University, College Station, TX 77843, USA

^2^Department of Chemical Engineering, Auburn University, Auburn, AL 36849, USA

^3^Department of Chemical Engineering, Prairie View University, Prairie View, TX 77446, USA

^4^Department of Mechanical Engineering, Prairie View University, Prairie View, TX 77446, USA

^5^Department of Materials Science & Engineering, Texas A&M University, College Station, TX 77843, USA

Corresponding Author: Prof. Abdoulaye Djire

Email: [adjire@tamu.edu](mailto:adjire@tamu.edu)


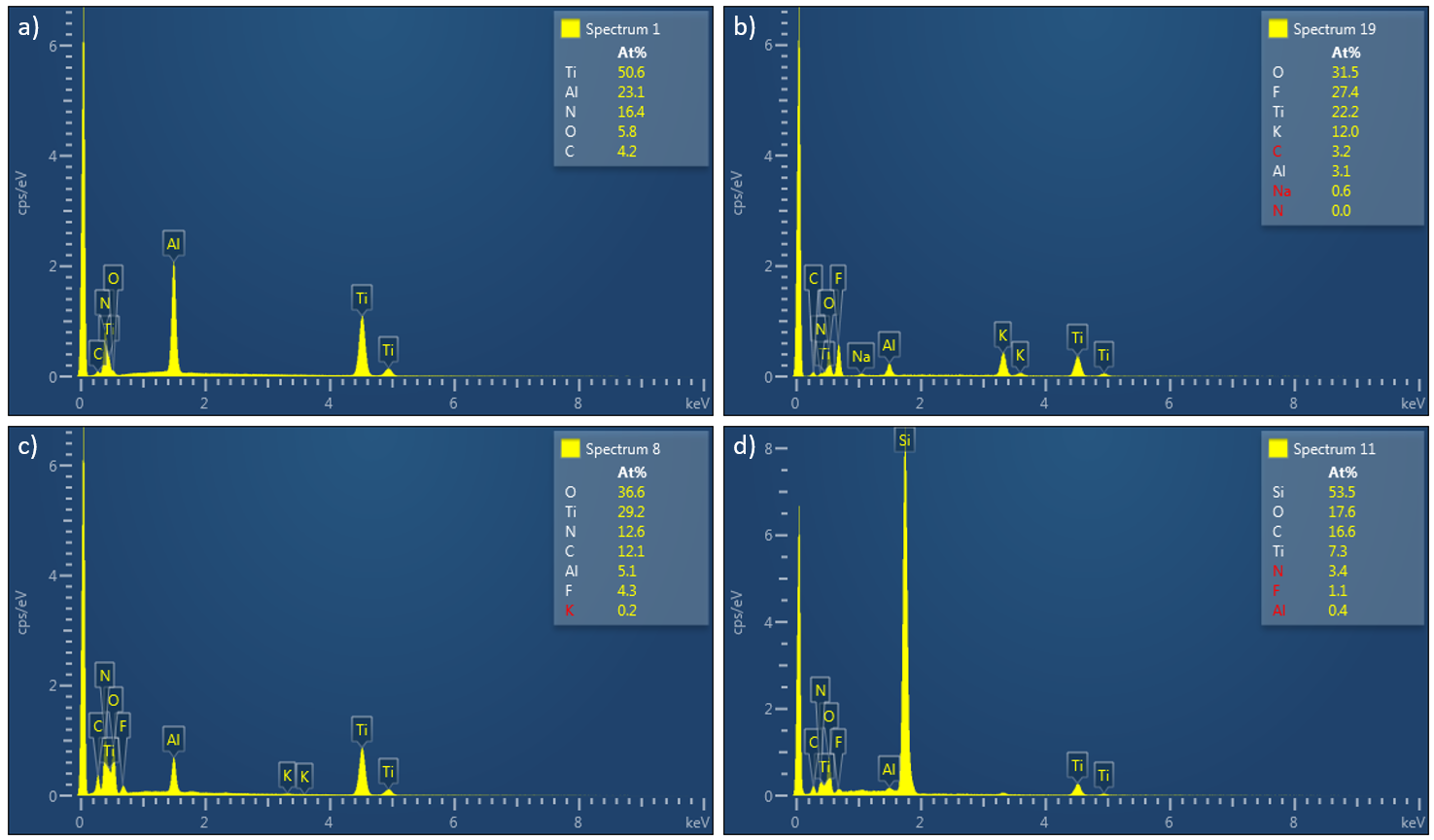


**Figure 1.** Electron Dispersive Spectroscopy (EDS) of **a.** Ti_2_AlN MAX phase, **b.** molten salt treated MAX phase, **c.** multilayer Ti_2_N MXene, and **d.** few-to-single layer Ti_2_N MXene. The large Si peak in the few-to-single layer sample is due to the substrate used for imaging.


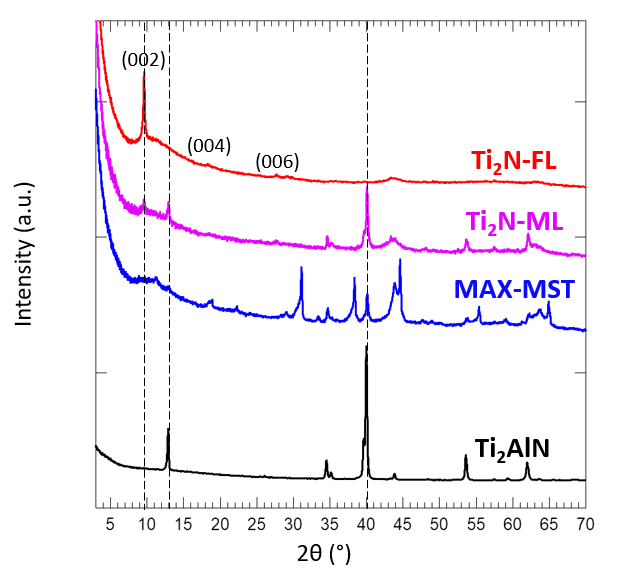


**Figure 2.** X-ray diffraction (XRD) spectroscopy of Ti_2_AlN MAX phase (black), molten salt treated MAX phase (blue), multilayer Ti_2_N MXene (magenta), and few-to-single layer Ti_2_N MXene (red). Spectra were gathered using a zero-diffraction silicon plate with a well.


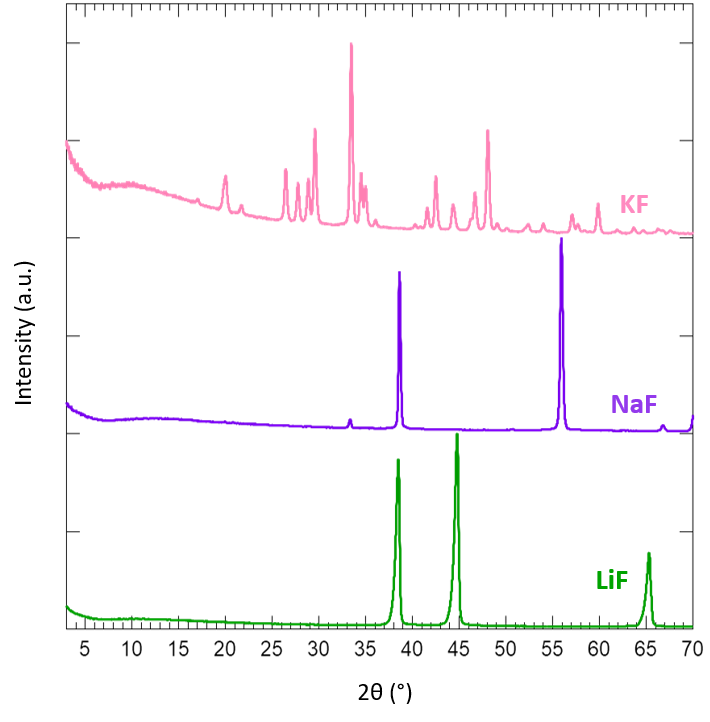


**Figure 3.** XRD spectroscopy of LiF (green), NaF (purple), and KF (pink) salts used in the oxygen-assisted molten salt treatment of MAX phase to form MXene.


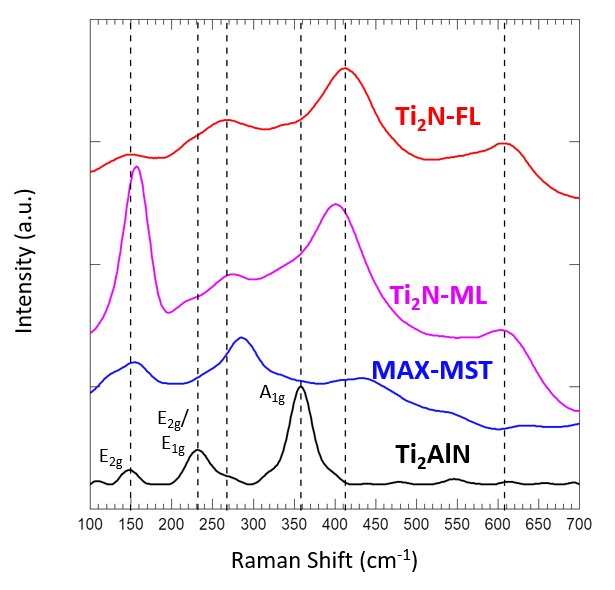


**Figure 4.** Raman spectroscopy of Ti_2_AlN MAX phase (black), molten salt treated MAX phase (blue), multilayer Ti_2_N MXene (magenta), and few-to-single layer Ti_2_N MXene (red). Spectra were collected using a 532 nm laser, 1800 lines/mm grating, and 100x objective lens.


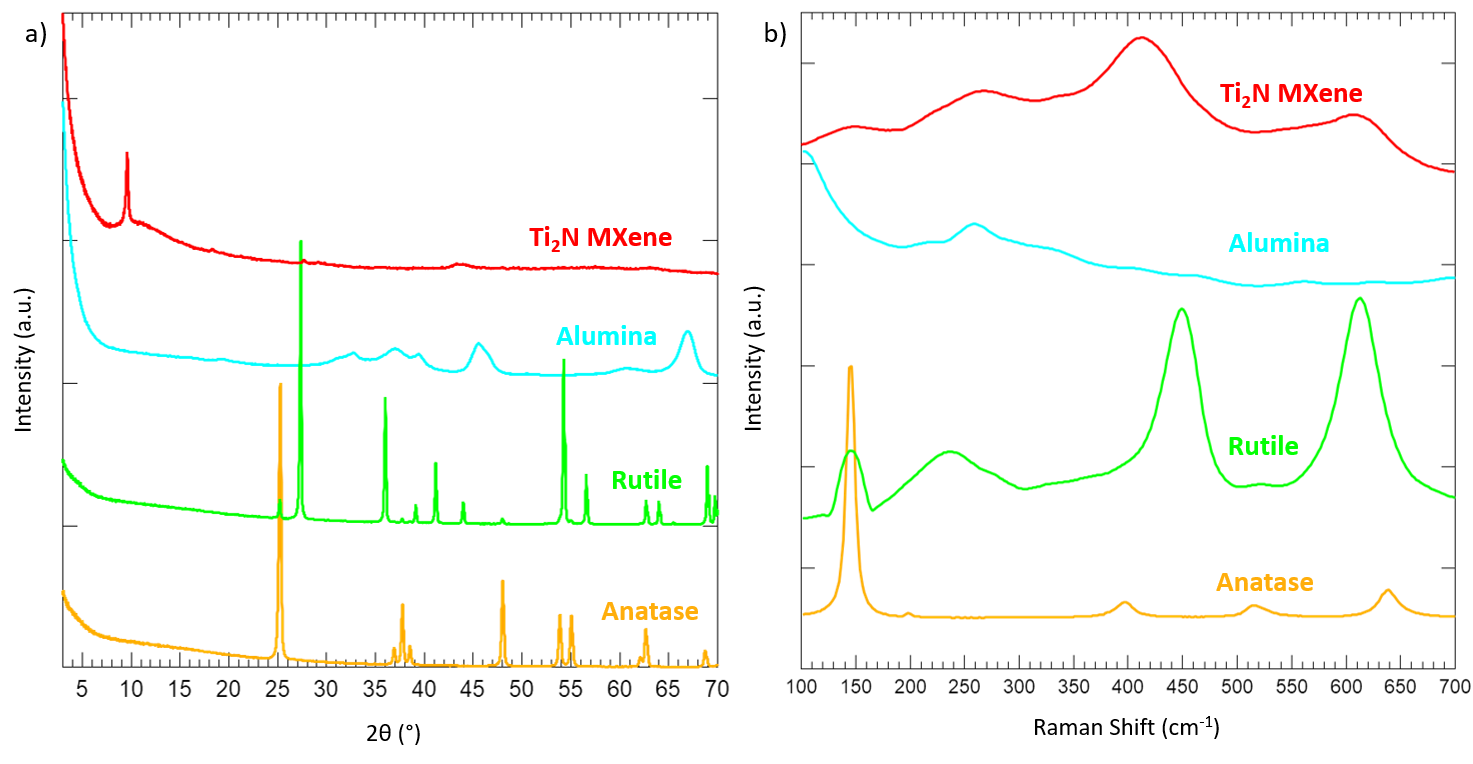


**Figure 5.** XRD and Raman spectroscopy of common oxide materials, Anatase (yellow), Rutile (green), and Alumina (light blue), encountered with the oxygen-assisted molten salt treatment of MAX phases.


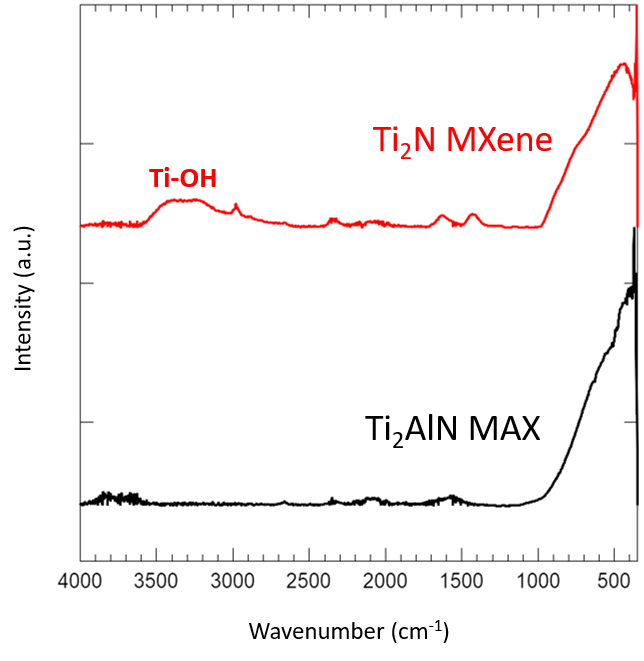


**Figure 6.** Fourier Transform Infrared (FTIR) spectroscopy of Ti_2_AlN MAX phase (black) and few-to-single layer Ti_2_N MXene to discern functional group existence. Analysis conducted using a diamond crystal ATR configuration.


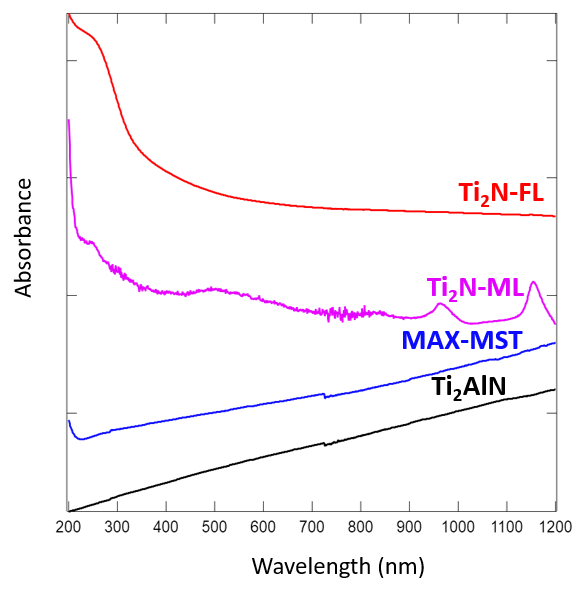


**Figure 7.** UV-Vis spectroscopy of Ti_2_AlN MAX phase (black), molten salt treated MAX phase (blue), multilayer Ti_2_N MXene (magenta), and few-to-single layer Ti_2_N MXene (red). Spectra collected using water as the reference.


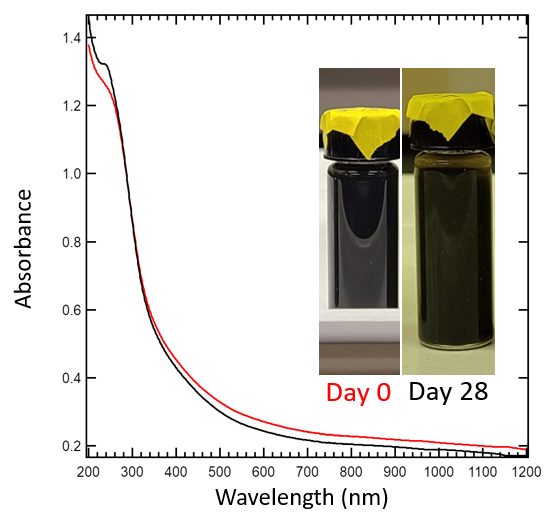


**Figure 8.** Analysis of aging effects through the use of UV-Vis spectroscopy and digital photography (inset) after 28 days.


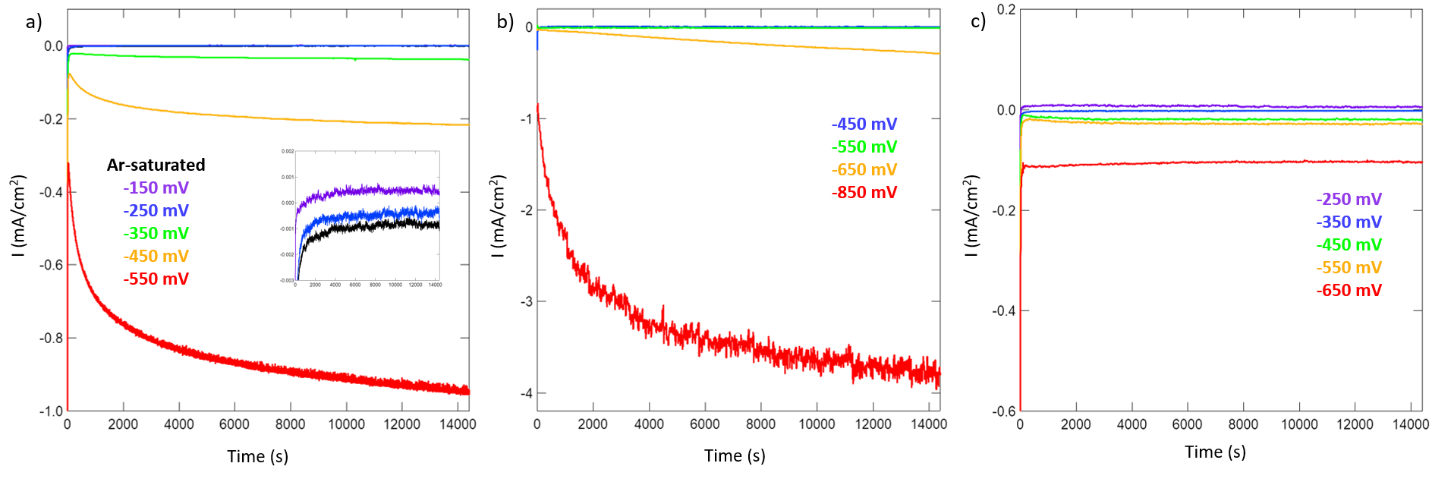


**Figure 9.** Chronoamperometry (CA) curves from 4-hour nitrogen reduction reaction experiments at varying reaction conditions with **a.** Ti_2_N, **b.** Ti_3_CN, and **c.** Ti_3_C_2_ as the electrocatalyst.


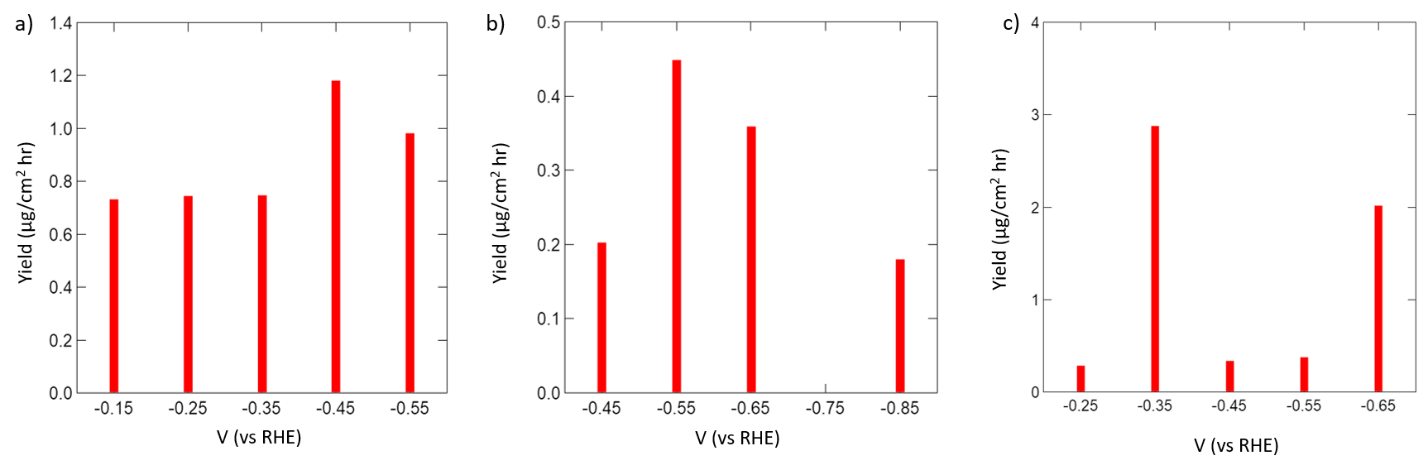


**Figure 10.** Hydrazine (N_2_H_4_) yield of **a.** Ti_2_N, **b.** Ti_3_CN, and **c.** Ti_3_C_2_ MXenes at differing potentials after 4-hour chronoamperometry experiments.


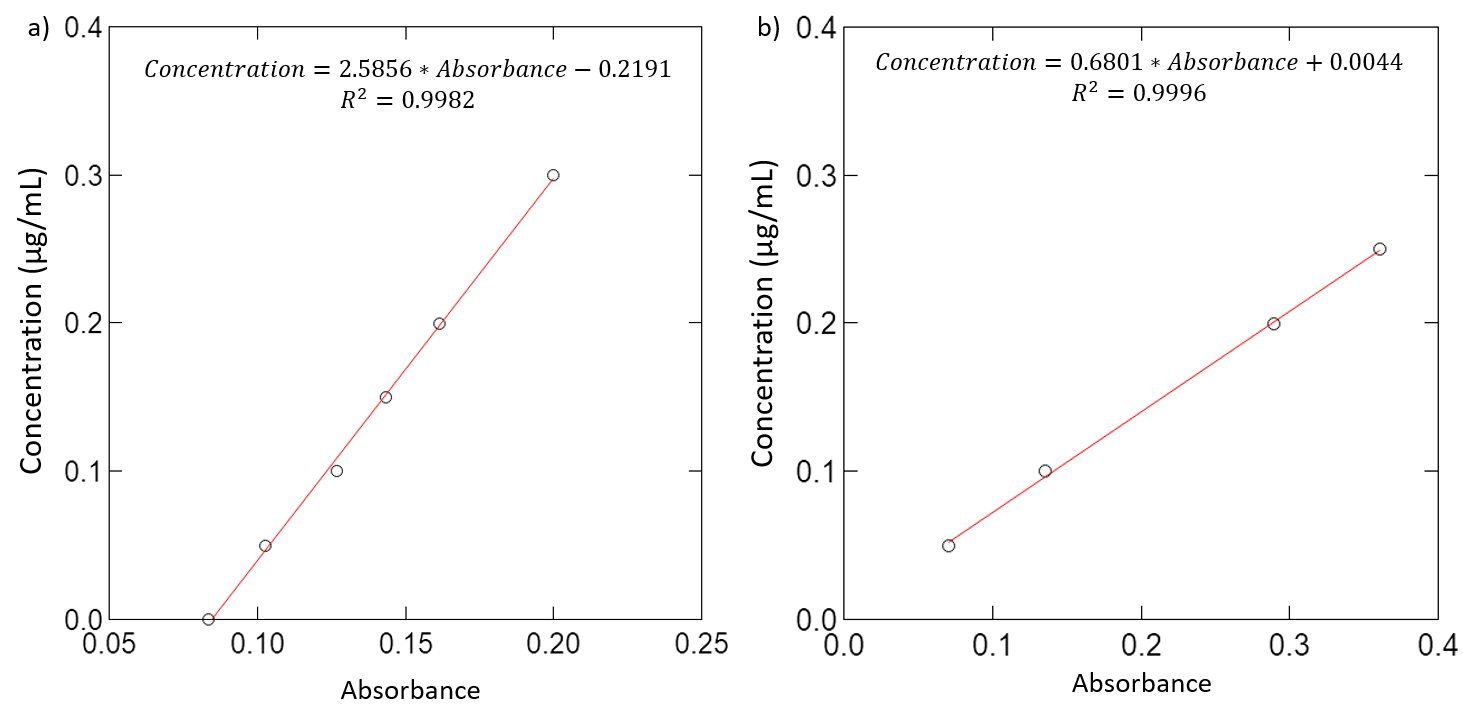


**Figure 11.** Calibration curves for determination of **a.** NH_3_ and **b.** N_2_H_4_ concentrations through the absorbance values gathered from UV-Vis spectroscopy after using the indophenol blue method and Watt-Chrisp method, respectively.


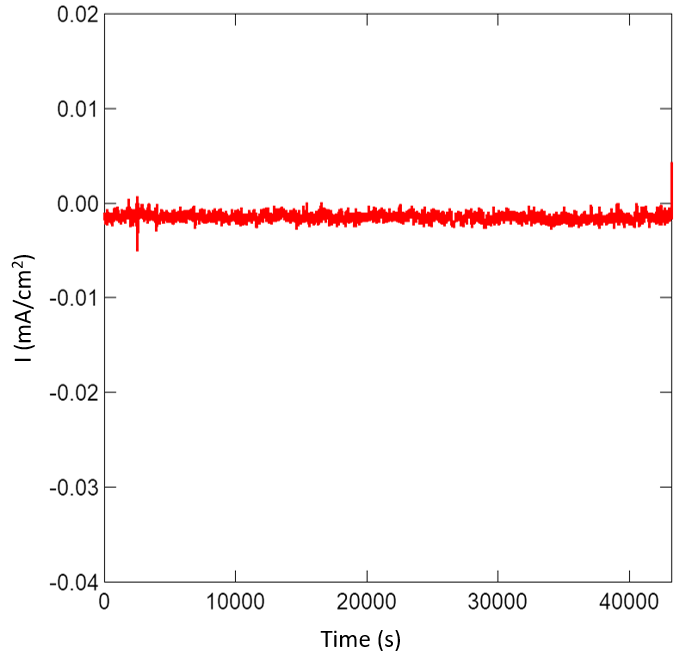


**Figure 12.** Chronoamperometry (CA) curve from a 12-hour nitrogen reduction reaction experiment conducted at –250 mV with Ti_2_N electrocatalyst.


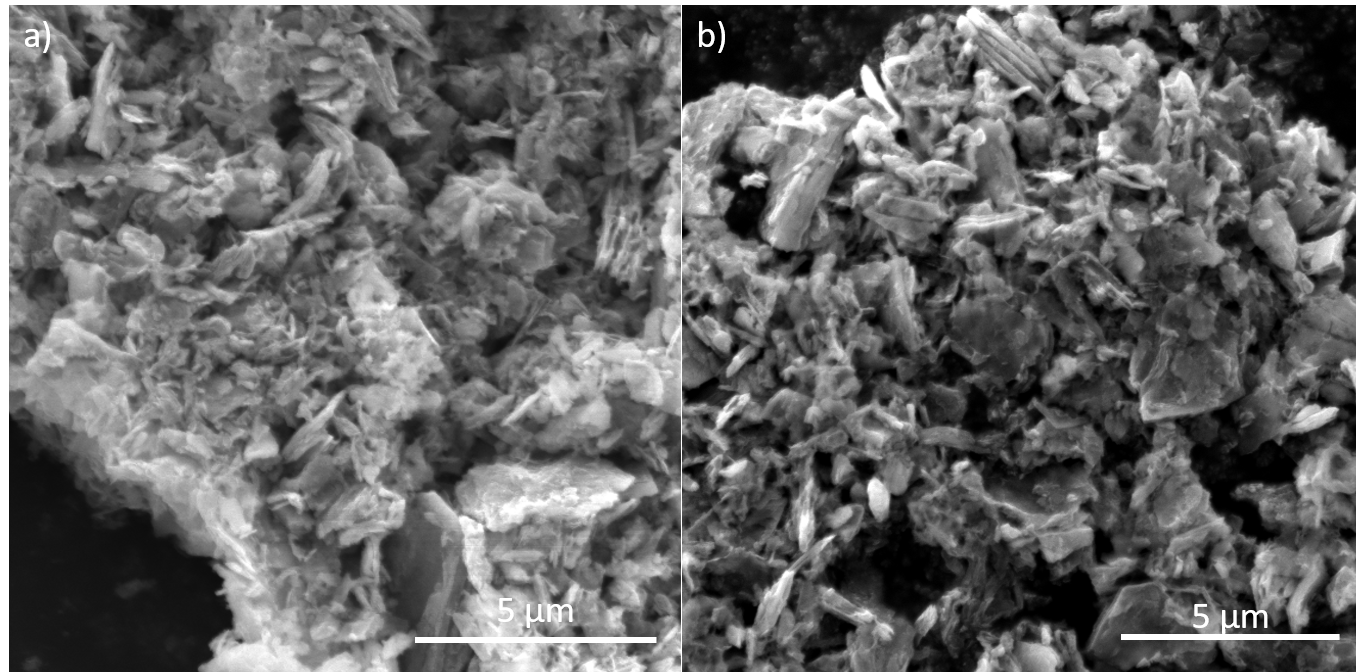


**Figure 13.** SEM imaging of drop-cast Ti_2_N transferred to carbon tape **a.** before and **b.** after being exposed to NRR conditions.


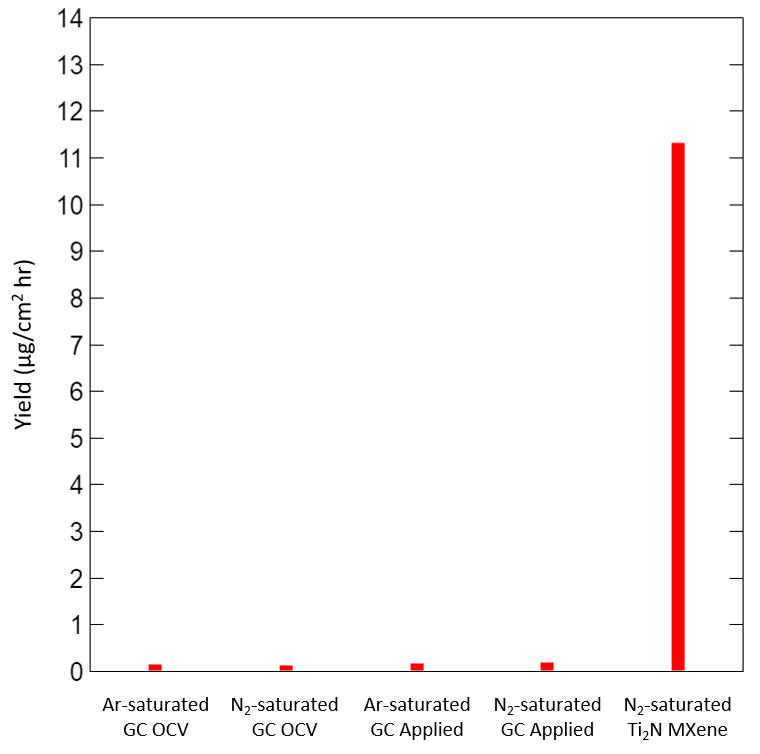


**Figure 14.** Ammonia (NH_3_) yield of control experiments. GC stands for the blank glassy carbon electrode that the electrocatalyst was prepared on. OCV stands for Open Circuit Voltage, meaning that no potential was applied to the system for the 4-hour period. Applied stands for having the highest performing NRR voltage (–250 mV) applied to the system for 4 hours. Values are compared against the reported values from this report.
